# Late Onset Myasthenia Gravis Is Associated with HLA DRB1*15:01 in the Norwegian Population

**DOI:** 10.1371/journal.pone.0036603

**Published:** 2012-05-09

**Authors:** Angelina H. Maniaol, Ahmed Elsais, Åslaug R. Lorentzen, Jone F. Owe, Marte K. Viken, Hanne Sæther, Siri T. Flåm, Geir Bråthen, Margitta T. Kampman, Rune Midgard, Marte Christensen, Anna Rognerud, Emilia Kerty, Nils Erik Gilhus, Chantal M. E. Tallaksen, Benedicte A. Lie, Hanne F. Harbo

**Affiliations:** 1 Department of Neurology, Oslo University Hospital, Ullevål, Oslo, Norway; 2 Department of Neurology, Oslo University Hospital, Rikshospitalet, Oslo, Norway; 3 Department of Immunology, Oslo University Hospital, Rikshospitalet, Oslo, Norway; 4 Department of Neurology, Haukeland University Hospital, Bergen, Norway; 5 Department of Neurology, St. Olavs Hospital, Trondheim, Norway; 6 Centre for clinical research and education, University Hospital of North Norway, Tromsø, Norway; 7 Department of Neurology, Molde Hospital, Molde, Norway; 8 Department of Neurology, Stavanger University Hospital, Stavanger, Norway; 9 Department of Neurology, Lillehammer Hospital, Lillehammer, Norway; 10 Department of Clinical Medicine, University of Bergen, Bergen, Norway; 11 Department of Medical Genetics, University of Oslo, Oslo, Norway; 12 Institute of Clinical Medicine, University of Oslo, Oslo, Norway; 13 Department of Clinical Medicine, University of Tromsø, Tromø, Norway; University of Alabama at Birmingham, United States of America

## Abstract

**Background:**

Acquired myasthenia gravis (MG) is a rare antibody-mediated autoimmune disease caused by impaired neuromuscular transmission, leading to abnormal muscle fatigability. The aetiology is complex, including genetic risk factors of the human leukocyte antigen (HLA) complex and unknown environmental factors. Although associations between the HLA complex and MG are well established, not all involved components of the HLA predisposition to this heterogeneous disease have been revealed. Well-powered and comprehensive HLA analyses of subgroups in MG are warranted, especially in late onset MG.

**Methodology/Principal Findings:**

This case-control association study is of a large population-based Norwegian cohort of 369 MG patients and 651 healthy controls. We performed comprehensive genotyping of four classical HLA loci (HLA-A, -B, -C and -DRB1) and showed that the DRB1*15:01 allele conferred the strongest risk in late onset MG (LOMG; onset ≥60years) (OR 2.38, p_c_7.4×10^−5^). DRB1*13:01 was found to be a protective allele for both early onset MG (EOMG) and LOMG (OR 0.31, p_c_ 4.71×10^−4^), a finding not previously described. No significant association was found to the DRB1*07:01 allele (p_nc_ = 0.18) in a subset of nonthymomatous anti-titin antibody positive LOMG as reported by others. HLA-B*08 was mapped to give the strongest contribution to EOMG, supporting previous studies.

**Conclusion:**

The results from this study provide important new information concerning the susceptibility of HLA alleles in Caucasian MG, with highlights on DRB1*15:01 as being a major risk allele in LOMG.

## Introduction

Acquired myasthenia gravis (MG) is a rare autoimmune neuromuscular disease with an overall prevalence of 10–20 per 100 000 [1]. MG is caused by impaired neuromuscular transmission leading to abnormal muscle fatigability affecting in some cases only the eye muscles (ocular MG), but in most cases several muscles groups (generalised MG) [2], [3]. The muscle fatigability is mediated by pathogenic autoantibodies against the muscle acetylcholine receptors (AChR-abs) detectable in the majority of patients (80–85%) [4]. Among the remaining patients without AChR-abs, 10–50% have antibodies to the muscle specific kinase (MuSK) [5], [6]. Recent studies have revealed that some might have low-affinity AChR-abs, to date not detectable with routinely used assays [7]. MG is characterized by remarkable heterogeneity, including degree of thymus involvement and clinical presentation like age at onset, disease severity and response to treatment [8].

The two major subgroups of patients are currently classified according to age at onset: early onset MG (EOMG) and late onset MG (LOMG). Age-cut off between these subgroups differs between studies, ranging from 40 and 50 years at onset [9]. Another MG subgroup consists of patients with thymoma, which is a paraneoplastic condition that occurs in 10–15% of all MG patients and at any age [10]. Typically, EOMG shows thymus hyperplasia and a strong female preponderance, while LOMG has a male predominance and normal or atrophic thymus findings [8]. LOMG is considered to be a more heterogeneous group than EOMG. Some LOMG patients with age at onset between 40 and 50 years might represent EOMG with delayed onset [11], [12]. A subset of LOMG with detectable anti-titin antibodies (ATA) in about 50% of the cases has also been reported, whereas ATA is rarely found in EOMG [13], [14]. Thus, to define a more homogeneous group of LOMG, some clinical studies have used 60 years as age cut-off [15].

The aetiology of MG is complex and explained by a combination of genetic and unknown environmental factors [16]. The genetic associations found in MG are several [17], and the most important genetic risk factor is conferred to the human leukocyte antigen (HLA) complex, as it is for many other autoimmune diseases [18]. The first genetic studies of Caucasian MG showed different associations to HLA alleles in both Class I (HLA-A, -B and -C) and Class II (HLA-DRB1 and -DQB1), suggesting that the heterogeneity of the disease may be explained partly on a genetic basis [19]–[26]. An increased prevalence of the extended HLA A1-B8-DR3 haplotype (also called the ancestral haplotype AH 8.1) was found in patients with disease onset before the age of 40 years i.e. EOMG, while an association with the HLA-B7-DR2 haplotype was reported in patients with onset age older than 40 years i.e. LOMG. MG with thymoma has consistently not shown associations with HLA, except for a recent study reporting a positive association with the HLA-A locus [27].

**Figure 1:**
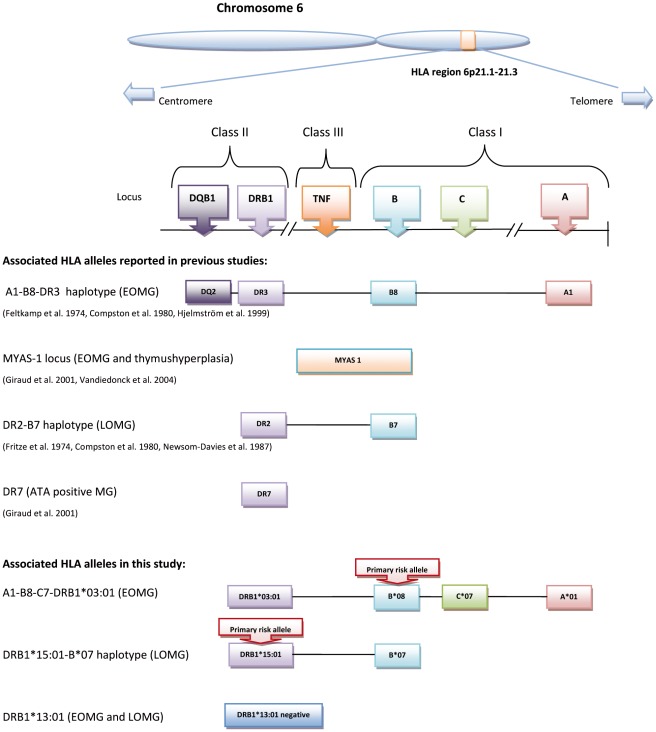
Schematic overview of associated HLA alleles in MG. The HLA complex on chromosome 6 with its division into three classes. Some key genes and their order on the chromosome are given. Associated HLA alleles reported earlier in Caucasian MG patients (see text for complete references) are illustrated together with the results from the present study.

Three decades ago, Compston and colleagues first addressed the different HLA genetic risk factors in EOMG and LOMG [23]. Since then, several studies have aimed to find the diseases causative locus in MG subgroups (Figure 1) [28]–[36], but the strong linkage disequilibrium (LD) in the HLA complex has long hampered this search [28], [29], [37]. There has also been lack of comprehensive HLA genotyping in population-based studies comprising a sufficient number of both EOMG and LOMG cases. The association with haplotype AH 8.1 in EOMG has been largely consistent, and several studies have suggested HLA-B8 as the predominant associated allele in this haplotype [30]–[32], [35]. In LOMG, the search for HLA risk alleles has been more challenging, partly because of limited sample sizes and the clinical heterogeneity within the subgroup. Although previous studies have pointed out HLA-B7 and/or DR2 as associated loci [23], [30], [37] and recently DR7 in a subset of nonthymomatous anti-titin antibodies (ATA) positive LOMG [35], definite HLA genetic risk factors for LOMG have not yet been established.

Based on this background, this study aimed to investigate a large Norwegian MG population with well defined subgroups of patients, and carry out comprehensive genotyping of HLA Class I and II loci in order to better characterise the genetic risk factors in MG subgroups In particular, we aimed to focus on finding the major HLA risk allele in the LOMG subgroup.

## Materials and Methods

### Patients and Controls

In this population-based study, subjects with acquired MG (≥16 years) were collected from all neurological departments and clinics with private neurologists in Norway in the period of 1.1.2005–1.11.2009. The inclusion criteria for acquired MG were at least “1 and 2” or “1 and 3” of the following criteria: 1) clinical MG, 2) neurophysiologic evidence consistent with MG (decrement >10% at 3 Hz after repetitive motor nerve stimulation, increased jitter on single-fibre electromyogram [38] or both), and 3) elevated level of acetylcholine receptor (AChR) antibodies or muscle specific kinase (MuSK) antibodies. Exclusion criteria were age <16 years, and lack of both criteria 2 and 3, mentioned above.

A total of 532 MG patients were identified, and 404 (76%) consented to participate in the genetic study by donating blood samples for DNA preparation. Retrospectively, clinical information from the study participants (n = 404) was obtained from the patient’s medical records, and antibodies titres were checked with the AChR-abs Register at the Department of Neurology, Haukeland University Hospital, and Bergen, Norway. Clinical information registered include: age at disease onset, AChR-abs, concomitant immune-mediated diseases, thymectomy, thymus histopathology and highest score of disease severity according to revised classification proposed by the Task Force of The Medical Advisory Board of the Myasthenia Gravis Foundation of America (MGFA) [39].

To minimize possible population stratification, all MG subjects included in the HLA case-control study were ethnically Norwegian, as were the control subjects. Hence, 26 non-ethnic Norwegian MG patients were excluded, among whom one was MuSK positive. Only one additional MuSK positive patient was recognised and also excluded. Altogether, 378 unrelated Norwegian Caucasian MG patients were included in the HLA case-control study.

The control samples (n = 651, female: male ratio 1.9) were randomly selected among healthy Norwegian bone marrow donors recruited through the Norwegian Bone Marrow Donor Registry (http:/www.nordonor.org/). The same control sample set has been used in other studies [40]. Written informed consent was obtained from all study participants. The study was approved by The Regional Committee for Research Ethics.

### Sub-classification of MG

The sub-classification of patients was done according to age at onset and presence or absence of thymoma. EOMG was defined as age at onset of 40 years or younger. Because of the uncertainty as to where to set the age-cut off for LOMG, as previously described by Aarli and colleagues [12], we classified MG with onset over 40 years into two groups: an “intermediate group” with age at onset of 41–59 years, and a late onset group (LOMG) with age at onset of 60 years or older.

### HLA Genotyping

Genomic DNA was extracted from peripheral blood lymphocytes using an in-house desalting method. HLA Class I (HLA-A, - B, and -C) genotypes of the MG patients were resolved to a consistent two-digit resolution level, and Class II (HLA-DRB1) to a four-digit resolution level, using in-house sequencing-based approaches. Ambiguities at DRB1 were resolved by selecting the most probable genotype based on allele frequencies reported in Caucasians (http://bioinformatics.nmdp.org/HLA). If the ambiguous genotypes had similar probabilities the genotype was assigned as missing. The genotyping method used is described elsewhere [41] and involves a polymerase chain reaction (PCR) amplification using the Platinum® Taq PCRx DNA polymerase kit (Invitrogen, Carlsbad, CA, USA) followed by sequencing of exon 2 and 3 (for Class I) and exon 2 (for Class II) using BigDye® Terminator v3.1 chemistry (Applied Biosystems, Foster City, CA, USA) and an ABI3730 capillary sequencer (Applied Biosystems). Primers and protocols used for sequencing of the individual HLA loci are available from the authors upon request. Allele assignment was performed using Assigns SBT® v3.2.7b (Conexio Genomics, Applecross, Australia) and the IMGT/HLA database v. 2.28.0 (http://www.ebi.ac.uk/imgt/hla/). HLA data for the healthy controls were available from the Norwegian Bone Marrow Donor Registry, and were at the same resolution level as the patients’.

### Statistical Analysis

Statistical analysis of genetic associations was performed using UNPHASED v.2.404-w32 [42]. Rare alleles (n<2 in patients and controls) were excluded. Conditional logistic regression (“main effects” test) was used to evaluate the dependency of the risk contribution of the different loci HLA-A, -B, -C and -DRB1 in EOMG in order to determine the most strongly associated locus. The expectation maximization algorithm was used to estimate maximum likelihood haplotype frequencies of B and DRB1 from the genotypes at each locus, assuming Hardy-Weinberg equilibrium. The haplotype method [43] and the Svejgaard method [44] were used to assess which alleles and loci showed the primary association and which appeared to be secondary due to LD. Values for D’ and r^2^ were calculated for allele combination. Odds ratios (OR) and 95% confidence intervals (CI) were calculated with Woolf’s formula with Haldane’s correction for nominal p-values (p_nc_). P-values <0.01 after correction for number of comparisons in the initial global locus tests (n = 4) were considered significant. For allelic associations, novel associations were corrected for number of comparisons before claiming significant associations. Only the number of tested alleles at each locus were corrected using Bonferroni correction (p_c_), as the alleles at HLA loci do not fully represent independent tests due to the strong LD (n = 8 for HLA-A, n = 21 for HLA-B, n = 13 for HLA-C and n = 38 for HLA-DRB1). For alleles previously published to be associated, uncorrected p-values (p_nc_) are also shown. When assessing the strongest associations by conditional analyses, no corrections were performed in order to avoid camouflaging weak effects due to LD.

## Results

A total of 369 Norwegian MG patients were successfully genotyped for all four loci genotyped; 9 patients were excluded due to missing genotypes and technical difficulties in genotyping. The clinical characteristics of the MG cohort investigated are shown in Table 1, and the associations between HLA loci and MG subgroups are shown in Table 2. There were significant associations of both the HLA Class I (HLA-A, -B and -C) loci and the Class II (HLA-DRB1) locus in the EOMG group. LOMG showed a strong association with only the DRB1 locus. No significant associations were observed in any HLA loci in the intermediate group (onset at 41–59 years) or to MG with thymoma. The most frequent haplotype was AH 8.1 in both EOMG (21%) and controls (9%), while HLA-A*02- or A*03-B*07-C*07-DRB1*15:01 haplotype was most frequent in LOMG (13%). An overview of allele frequencies in MG subgroups and controls is available as supporting information in Table S1. The clinical characteristics of MG patients carrying the most strongly associated HLA genotypes reported in this study are shown in Table S2 as supporting information.

**Table 1:** Clinical characteristics of the Norwegian myasthenia gravis study cohort (n = 369).

MG subgroups	Totaln = 369, n (%)	Femalen = 229, n (%)	AChR-ab+n = 300, n (%)	Thymectomyn = 168, n (%)	Ocular MG^1^n = 68, n (%)	Immune-mediated diseases^2^n = 110, n (%)
EOMG (≤40)	154 (42)	131 (85)	116 (75)	108 (70)	20 (13)	49 (32)
MG with onset 41−59 years	86 (23)	45 (52)	60 (70)	26 (30)	30 (35)	25 (29)
LOMG (≥60)	99 (27)	39 (39)	94 (95)	5 (5)	16 (16)	31 (31)
Thymoma	30 (8)	14 (47)	30 (100)	29 (97)	3 (10)	5 (17)

1 According to MGFA classification [39]: ocular MG (MGFA grade I), generalised MG (MGFA grade II-V), not available in 12 cases.

2 Concomitant immune-mediated diseases include thyroid disease (hypo-, hyperthyroidsm, thyroiditis), type 1-diabetes, rheumatic diseases, systemic lupus erythematosus (SLE), celiac disease, inflammatory bowel diseases (Crohńs disease or ulcerative colitits). EOMG = early onset MG; LOMG = late onset MG; AChR-ab+  =  acetylcholine receptor-antibody positive.

**Table 2:** The association between HLA loci and myasthenia gravis subgroups (p-values).

Locus	EOMG n = 154	MG with onset 41–59 years,n = 86	LOMG (≥60 years) n = 99	Thymoman = 30
HLA-A	2.1×10^−8^	0.44	0.18	0.37
HLA-B	9.0×10^−10^	0.41	0.04	0.56
HLA-C	4.4×10^−5^	0.21	0.77	0.92
HLA-DRB1	4.2×10^−10^	0.14	1.2×10^−6^	0.49

EOMG = early onset MG, LOMG = late onset MG.

P-values are not corrected, p<0.01 is considered significant after correction for multiple comparisons (n = 4).

### LOMG

The DRB1 locus showed a strong association to LOMG (p_nc_ = 1.2.5×10^−6^), while the B-locus was not associated (p_nc_ = 0.04). The strongest associated alleles in LOMG are shown in Table 3, pointing to the DRB1*15:01 allele conferring the strongest risk (OR 2.38, p_nc_ = 2.0×10^−^6, p_c_ = 7.4×10^−^5). Interestingly, other DR2 alleles, e.g. DRB1*15:02 were not associated (p_nc_ = 0.11). The DRB1*03:01 (p_nc_ = 0.001, p_c_ = 0.05) and DRB1*13:01(p_nc_ = 0.004, p_c_ = 0.15) alleles showed modest novel negative associations, however, when correcting for number of comparisons (n = 38) in this analysis, these associations were no longer significant, as also observed for the negative association with B*08 (p_nc_ = 0.003, p_c_ = 0.07). The previously reported association to HLA-B7 in LOMG was only weakly present in our study (p_nc_ = 0.01). Haplotype analysis of the DRB1*15:01 versus other HLA-B alleles showed the strongest LD with B*07 (D’ = 0.5, r^2^ = 0.2). This might explain the borderline association found to B*07, and support that DRB1*15:01 is the superior risk allele in LOMG. Sub-analysis of the nonthymomatous ATA positive late onset MG patients over 40 years (n = 47) did not show significant association with the DRB1*07:01 allele (p_nc_ = 0.18).

**Table 3:** Associated alleles in late onset myasthenia gravis (LOMG).

Allele	Allele frequencyCases n = 99	Allele frequencyControls n = 651	OR	95% CI	Nominalp-value	Bonferroni correctedp-value
B*07	0.21	0.15	1.61	1.04−1.90	0.01	0.21
B*08	0.06	0.13	0.42	0.23−0.75	0.003	0.06
DRB1*03:01	0.05	0.13	0.35	0.19−0.67	0.001	0.04
DRB1*13:01	0.01	0.07	0.22	0.08−0.62	0.004	0.15
DRB1*15:01	0.26	0.13	2.38	1.66−3.40	2.0×10^−6^	7.6×10^−5^

Odds Ratio (OR) and 95% confidence interval (CI) are shown for nominal p-values.

In EOMG, positive associations were observed for the well-established HLA-A*01, -B*08, -C*07 and -DRB1*0301 alleles (Table 4). Conditional tests showed that virtually all associations at the other loci disappeared when conditioning on either HLA-B or -DRB1 (Table 5), indicating that these two loci are stronger determinants than HLA-A and -C. In order to assess which of the positively associated alleles at these two loci displayed the strongest association, we performed a conditional haplotype analysis of HLA-B*08 and -DRB1*03:01. Strong LD between the two alleles was confirmed (D’ = 0.8, r^2^ = 0.7). HLA-B*08 was found to confer risk to EOMG both when carried on the same haplotype as DRB1*03:01 (p = 0.037) and in the absence of DRB1*03:01 (p = 0.005), while DRB1*03:01 was not associated after stratifying the haplotypes according to B*08 (Table 6). Novel negative associations were seen with HLA-A*02 (p_nc_ = 4.6×10^−4^, p_c_ = 3.7×10^−3^), C*05 (p_nc_ = 4.6×10^−3^, p_c_ = 0.06) and DRB1*13:01(p_nc_ = 3.1×10^−3^, p_c_ = 0.1), however, only A*02 was significant associated after correction. This negative association of the most common HLA-A allele could be explained by a reduction in A*02 among patients expressing the protective A*01/B*08 haplotype. Hence, conditional haplotype analysis of HLA-A*02 and -B*08 was performed, and the results showed that HLA-A*02 did not confer significant protection when carried on the same haplotype as B*08 (p = 0.51) nor did it do so when carried on haplotypes without B*08 (p = 0.097) (data not shown).

**Table 4:** The most strongly associated HLA alleles in early onset myasthenia gravis (EOMG).

Allele	Allele frequencyCases n = 154	Allele frequencyControls n = 651	OR	95% CI	Nominalp-value	Bonferroni correctedp-value
A*01	0.31	0.16	2.43	1.83−3.22	6.1×10^−10^	4.9×10^−9^
A*02	0.24	0.35	0.60	0.45−0.80	4.6×10^−4^	3.7×10^−3^
B*08	0.33	0.13	3.12	2.30−4.20	1.2×10^−14^	2.5×10^−13^
C*07	0.47	0.32	1.90	1.48−2.45	5.8×10^−7^	7.5×10^−6^
C*05	0.05	0.10	0.45	0.26−0.78	4.6×10^−3^	0.06
DRB1*03:01	0.31	0.13	2.90	2.17−3.87	5.4×10^−13^	2.1×10^−11^
DRB1*07:01	0.03	0.09	0.39	0.21−0.72	2.7×10^−3^	0.10
DRB1*13:01	0.03	0.07	0.35	0.17−0.70	3.1×10^−3^	0.12

Odds Ratio (OR) and 95% confidence interval (CI) are shown for nominal p-values.

**Table 5:** Conditional analyses of the HLA-A, -B, -C and DRB1 loci (p-values) in early onset myasthenia gravis (EOMG).

Conditional test HLA loci				
**Locus**	**HLA-A**	**HLA-B**	**HLA-C**	**HLA-DRB1**
HLA-A	−	0.2	2.5×10^−5^	0.08
HLA-B	2.0×10^−4^	−	4.0×10^−4^	0.05
HLA-C	9.0×10^−4^	0.42	−	0.26
HLA-DRB1	2.0×10^−3^	0.09	8.0×10^−3^	−

P-values are not corrected, p<0.01 is considered significant after correction for multiple comparisons (n = 4).

**Table 6:** Assessment of the strongest association on the HLA-B*08-DRB1*03:01 haplotype in early onset myasthenia gravis (EOMG).

Comparison of haplotypes	P-value	OR	95% CI
Association of B*08+ vs. B*08−	1.0×10^−13^	3.01	2.01−4.02
Association of DRB1*03:01+ vs. DRB1*03:01−	2.7×10^−11^	2.69	2.01−3.60
Association of B*08 on DRB1*03:01+	3.7×10^−2^	2.15	1.04−4.42
Association of B*08 on DRB1*03:01−	2.5×10^−3^	2.61	1.40−4.87
Association of DRB1*03:01 on B*08+	0.56	1.22	0.63−2.36
Association of DRB1*03:01 on B*08−	0.26	1.49	0.75−2.95

+  =  positives, −  =  negatives.

To test whether the suggested negative association of HLA-DRB1*13:01 found in both EOMG and LOMG could be a general protective factor for MG, we pooled all MG subgroups (except MG with thymoma) and compared the groups with controls. This showed that the DRB1*13:01 allele was significantly associated with MG (OR 0.31, 95% CI 0.18–0.52, p_c_ = 4.71×10^−4^). To elaborate on the results showing that B*08/DRB1*03:01 are hazardous to EOMG and weakly protective to LOMG, we compared the allele frequency of both alleles between EOMG and LOMG subgroups. A significantly differential distribution between EOMG and LOMG was seen for both B*08 (p_c_ = 2.95×10^−9^), and also DRB1*03:01 (p_c_ = 2.56×10^−5^).

## Discussion

We have investigated the HLA associations of Class I and Class II alleles by performing comprehensive genotyping in a large and representative Norwegian MG population, and explored potential risk alleles in the different MG subgroups. Genetic differences between EOMG and LOMG have been reported before, but our study was able to define the primary risk alleles in EOMG and LOMG to be HLA-B*08 and HLA-DRB1*15:01, respectively, in our population. We also identified HLA-DRB1*13:01 as a general MG protective allele that confers protection to both EOMG and LOMG, a finding not reported before.

In LOMG, no genetic risk factors have been consistently described previously, but associations with HLA- B7 and/or -DR2 and DR 7 have been reported [23], [35]. A possible explanation for the divergent results may be that the cut-off age at onset used for LOMG has varied and that LOMG subgroups have been heterogeneous [11], [35]. By using age cut-off at 60 years for LOMG in this study, as suggested also by others [15], we were able to investigate a homogenous LOMG group. In doing so, we identified a significant association to the HLA-DR2 allele DRB1*15:01, which has not previously been reported by others. This allele is known to be quite common in particular European and Asian populations and is also the main genetic risk factor in another autoimmune neurological disease, i.e. multiple sclerosis [45]. It is, however, not known whether the occurrence of LOMG is lower in other MG populations of African or Amerindian origin where DRB1*15:01 is less frequent.

The DRB1 locus in LOMG has been examined in a previous family-based study of a large French MG cohort, but neither of the two DR2 alleles, DR15 or DR16, were found to be associated in a subset of LOMG comprising predominantly of males with normal thymus histopathology [35]. Instead, a subset of ATA positive LOMG was significantly associated with HLA-DR7 and negatively associated with DR3. This could not be reproduced in the subset of ATA positive LOMG in our Norwegian MG cohort. A borderline negative association of DRB1*03:01 was seen in the overall group of LOMG in our cohort, but no association was observed for DRB1*07:01. The lack of concordance between the results from LOMG in our cohort and the French MG cohort may be due partly to different inclusion criteria and genotyping methods. Further, a population specific association to DRB1*15:01 in the Norwegian MG population cannot be ruled out.

Due to the limited number of MG with thymoma (n = 30), the statistical power to detect the HLA-A association recently reported by others [27] may have been too low in our study. Only 8% had thymoma, which is lower than expected in a representative MG population [10]. However, strong HLA associations have not been reported in previous studies, and the susceptibility of MG patients with thymoma may be more likely explained by a paraneoplastic aetiology of the disorder [46].

For EOMG, the strongest predisposition observed in our study was for the HLA-B*08 allele. Previous studies have suggested the causative loci to be close to the Class III and Class I region of the HLA complex, pointing out HLA-B as a more superior risk locus than HLA-DRB1 [30], [32]. However, many of these studies were conducted at a time when genomic HLA typing was not available, and the knowledge of the genetic diversity of the HLA complex was more limited than today. It was therefore important to readdress this in our large MG study cohort using the higher resolution methods for HLA genotyping. In spite of the strong LD on the extended HLA -A1-B8-DR3 haplotype, we were able to map the primary HLA association to the B*08 allele.

Our results underline a clear difference in the association of B*08/DRB1*03:01 genotype between EOMG and LOMG, especially between EOMG with age at onset below 40 years and late onset MG with disease onset after 60 years. The intermediate group of late onset MG with age at onset between 41–59 years appears to be an overlapping group of MG, with a mixture of EOMG and LOMG genetic risk factors. Based on these results, it is still difficult to determine if the subset of LOMG with age of onset 41–59 years belongs to an EOMG or a LOMG subphenotype. This suggests that disease management and treatment of these MG patients should still be evaluated individually, as also implied by others [11], [47].

The main strengths of our study are the large sample of EOMG and LOMG patients from one population, extensive clinical measures of the study cohort, and the use of comprehensive HLA genotypin. However, there are also some limitations. As we did not genotype the HLA-DQB1 and DQA1 loci, which are in strong LD with the DRB1 locus, we cannot rule out that one of these (or in fact other surrounding loci in LD) is the primary risk locus. Both the haplotypes DRB*15:01- DQB1*06:02 and DRB*13:01-DQB1*06:03 are inherited together in our Norwegian population, which would hamper our ability to pinpoint the causal locus also in the presence of full class II genotyping data. We also note that the clinical characteristics of patients (e.g. thymus histopathology and MGFA scores) were reported by different clinicians from the collaborating neurological departments, and could have biased the classification of subgroups. Further, the method used for correcting p-values, using Bonferroni correction only within each locus, may be too anti-conservative. However, due to the strong LD between the loci, a more conservative method could overestimate p-values and camouflage novel associations. Therefore, weak associations reported in this study need to be interpreted with caution. Nevertheless, HLA-DRB1*13:01 was weakly negative associated with both EOMG and LOMG separately, but was highly significant when the total MG population (excluding MG with thymoma) was investigated. Because MG is a rare and also a heterogeneous disease, low sample sizes of the different MG subgroups have long hampered genetic studies of MG. Thus, for future investigation of genetic risk factors in MG, collaborative networks will be essential.

To the best of our knowledge, this study with comprehensive genotyping of four HLA loci in MG is the first to provide detailed HLA data in a large sample of MG patients comprising both EOMG and LOMG subtypes. The HLA alleles also continue to play a leading role in the genetic risk in other complex autoimmune diseases such as type-1 diabetes, multiple sclerosis and rheumatoid arthritis [18]. Even in the present time of genome-wide association studies (GWAS), well-designed studies dissecting the nature of the HLA associations in subgroups of MG patients are much needed.

In conclusion, this study provides both novel and supporting information of major HLA-associations in Caucasian MG. The novelties of our findings are the identification of DRB1*15:01 as the strongest HLA risk allele in LOMG, and the finding of DRB1*13:01 being a general protective allele for MG in our population. Furthermore, HLA-B*08 was mapped to be the primary risk allele in EOMG, underlining the distinct different genetic HLA profile in EOMG and LOMG subgroups. This needs to be taken into consideration in further studies investigating risk factors in MG.

## Supporting Information

Table S1 **Allele frequencies in MG subgroups and controls.** Allele groups that include rare alleles are: HLA-A*09 (23, 24), A*10 (25, 26, 34, 66), A*19 (29, 30, 31, 32, 33), A*28 (68, 69), B*05 (51, 52), B*12 (44, 45), B*15 (62), B*16 (38, 39), B*17 (57, 58), B*21 (49, 50), B*22 (54, 55, 56), B*40 (60, 61).
(DOC)

Table S2 **Clinical characteristics of the MG study cohort (n = 339) in relation to the most strongly associated HLA genotypes of this study.** ^1^According to MGFA classification [39]: ocular MG (MGFA grade I), generalised MG (MGFA grade II-V), not available in 12 cases. ^2^Thymus histopathology: hyperplasia or normal/atrophy, not available in 26 cases. ^3^Concomitant immune-mediated diseases in patients include thyroid disease (hypo-, hyperthyroidsm, thyroiditis), type 1-diabetes, rheumatic diseases, systemic lupus erythematosus (SLE), celiac disease, inflammatory bowel diseases (Crohńs disease or ulcerative colitits). *MG patients with thymoma (n = 30) were excluded. −  =  positives, −  =  negatives.
(DOC)

## References

[1] Carr AS, Cardwell CR, McCarron PO, McConville J (2010). A systematic review of population based epidemiological studies in Myasthenia Gravis..

[2] Oosterhuis HJ (1981). Myasthenia gravis.. Clin Neurol Neurosurg.

[3] Osserman KE, Genkins G (1971). Studies in myasthenia gravis: review of a twenty-year experience in over 1200 patients.. Mt Sinai J Med.

[4] Lindstrom JM, Seybold ME, Lennon VA, Whittingham S, Duane DD (1976). Antibody to acetylcholine receptor in myasthenia gravis. Prevalence, clinical correlates, and diagnostic value.. Neurology.

[5] Hoch W, McConville J, Helms S, Newsom-Davis J, Melms A (2001). Auto-antibodies to the receptor tyrosine kinase MuSK in patients with myasthenia gravis without acetylcholine receptor antibodies.. Nat Med.

[6] Vincent A, Bowen J, Newsom-Davis J, McConville J (2003). Seronegative generalised myasthenia gravis: clinical features, antibodies, and their targets.. Lancet Neurology.

[7] Leite MI, Jacob S, Viegas S, Cossins J, Clover L (2008). IgG1 antibodies to acetylcholine receptors in ‘seronegative’ myasthenia gravis.. Brain.

[8] Meriggioli MN, Sanders DB (2009). Autoimmune myasthenia gravis: emerging clinical and biological heterogeneity.. Lancet Neurol.

[9] Aarli JA (1999). Late-onset myasthenia gravis: a changing scene.. Arch Neurol.

[10] Evoli A, Minisci C, Di SC, Marsili F, Punzi C (2002). Thymoma in patients with MG: characteristics and long-term outcome.. Neurology.

[11] Aarli JA, Romi F, Skeie GO, Gilhus NE (2003). Myasthenia gravis in individuals over 40.. Ann N Y Acad Sci.

[12] Aarli JA (2008). Myasthenia gravis in the elderly: Is it different?. Ann N Y Acad Sci.

[13] Romi F, Skeie GO, Aarli JA, Gilhus NE (2000). Muscle autoantibodies in subgroups of myasthenia gravis patients.. J Neurol.

[14] Yamamoto AM, Gajdos P, Eymard B, Tranchant C, Warter JM (2001). Anti-titin antibodies in myasthenia gravis: tight association with thymoma and heterogeneity of nonthymoma patients.. Arch Neurol.

[15] Evoli A, Batocchi AP, Minisci C, Di SC, Tonali P (2000). Clinical characteristics and prognosis of myasthenia gravis in older people.. J Am Geriatr Soc.

[16] Vincent A (1994). Aetiological factors in development of myasthenia gravis.. Adv Neuroimmunol.

[17] Giraud M, Vandiedonck C, Garchon HJ (2008). Genetic factors in autoimmune myasthenia gravis.. Ann N Y Acad Sci.

[18] International MHC, Autoimmunity Genetics Network, Rioux JD, Goyette P, Vyse TJ, Hammarstrom L (2009). Mapping of multiple susceptibility variants within the MHC region for 7 immune-mediated diseases.. Proc Natl Acad Sci U S A.

[19] Feltkamp TE, van den Berg-Loonen PM, Nijenhuis LE, Engelfriet CP, van Rossum AL (1974). Myasthenia gravis, autoantibodies, and HL-A antigens.. Br Med J.

[20] Hammarstrom L, Smith E, Moller E, Franksson C, Matell G (1975). Myasthenia gravis: studies on HL-A antigens and lymphocyte subpopulations in patients with myasthenia gravis.. Clin Exp Immunol.

[21] Fritze D, Herrman C, Naeim F, Smith GS, Walford RL (1974). HL-A antigens in myasthenia gravis.. Lancet.

[22] van den Berg-Loonen EM, Nijenhuis LE, Engelfriet CP, Feltkamp TE, van Rossum AL (1977). Segregation of HLA haplotypes in 100 families with a myasthenia gravis patient.. J Immunogenet.

[23] Compston DA, Vincent A, Newsom-Davis J, Batchelor JR (1980). Clinical, pathological, HLA antigen and immunological evidence for disease heterogeneity in myasthenia gravis.. Brain.

[24] Newsom-Davis J, Willcox N, Schluep M, Harcourt G, Vincent A (1987). Immunological heterogeneity and cellular mechanisms in myasthenia gravis. Ann N Y Acad Sci..

[25] Kida K, Hayashi M, Yamada I, Matsuda H, Yoshinaga J (1987). Heterogeneity in myasthenia gravis: HLA phenotypes and autoantibody responses in ocular and generalized types.. Ann Neurol.

[26] Pirskanen R (1976). Genetic associations between myasthenia gravis and the HL-A system.. Journal of Neurology, Neurosurgery & Psychiatry.

[27] Vandiedonck C, Raffoux C, Eymard B, Tranchant C, Dulmet E (2009). Association of HLA-A in autoimmune myasthenia gravis with thymoma.. J Neuroimmunol.

[28] Vieira ML, Caillat-Zucman S, Gajdos P, Cohen-Kaminsky S, Casteur A (1993). Identification by genomic typing of non-DR3 HLA class II genes associated with myasthenia gravis.. J Neuroimmunol.

[29] Hjelmstrom P, Giscombe R, Lefvert AK, Pirskanen R, Kockum I (1995). Different HLA-DQ are positively and negatively associated in Swedish patients with myasthenia gravis.. Autoimmunity.

[30] Janer M, Cowland A, Picard J, Campbell D, Pontarotti P (1999). A susceptibility region for myasthenia gravis extending into the HLA-class I sector telomeric to HLA-C.. Hum Immunol.

[31] Degli-Esposti MA, Andreas A, Christiansen FT, Schalke B, Albert E (1992). An approach to the localization of the susceptibility genes for generalized myasthenia gravis by mapping recombinant ancestral haplotypes.. Immunogenetics.

[32] Vandiedonck C, Beaurain G, Giraud M, Hue-Beauvais C, Eymard B (2004). Pleiotropic effects of the 8.1 HLA haplotype in patients with autoimmune myasthenia gravis and thymus hyperplasia.. Proc Natl Acad Sci U S A.

[33] Machens A, Loliger C, Pichlmeier U, Emskotter T, Busch C (1998). The impact of HLA on long-term outcome after thymectomy for myasthenia gravis.. J Neuroimmunol.

[34] Carlsson B, Wallin J, Pirskanen R, Matell G, Smith CI (1990). Different HLA DR-DQ associations in subgroups of idiopathic myasthenia gravis.. Immunogenetics.

[35] Giraud M, Beaurain G, Yamamoto AM, Eymard B, Tranchant C (2001). Linkage of HLA to myasthenia gravis and genetic heterogeneity depending on anti-titin antibodies.. Neurology.

[36] Spurkland A, Gilhus NE, Ronningen KS, Aarli JA, Vartdal F (1991). Myasthenia gravis patients with thymus hyperplasia and myasthenia gravis patients with thymoma display different HLA associations.. Tissue Antigens.

[37] Machens A, Loliger C, Pichlmeier U, Emskotter T, Busch C (1999). Correlation of thymic pathology with HLA in myasthenia gravis.. Clin Immunol.

[38] Keesey JC (2004). Clinical evaluation and management of myasthenia gravis. [Review] [166 refs].. Muscle & Nerve.

[39] Jaretzki A, Barohn RJ, Ernstoff RM, Kaminski HJ, Keesey JC (2000). Myasthenia gravis: recommendations for clinical research standards. Task Force of the Medical Scientific Advisory Board of the Myasthenia Gravis Foundation of America. [Review] [51 refs].. Neurology.

[40] Lorentzen AR, Karlsen TH, Olsson M, Smestad C, Mero IL (2009). Killer immunoglobulin-like receptor ligand HLA-Bw4 protects against multiple sclerosis.. Ann Neurol.

[41] Sayer DC, Whidborne R, De SD, Rozemuller EH, Christiansen FT (2004). A multicenter international evaluation of single-tube amplification protocols for sequencing-based typing of HLA-DRB1 and HLA-DRB3,4,5.. Tissue Antigens.

[42] Dudbridge F (2003). Pedigree disequilibrium tests for multilocus haplotypes.. Genet Epidemiol.

[43] Valdes AM, Thomson G (1997). Detecting disease-predisposing variants: the haplotype method.. Am J Hum Genet.

[44] Svejgaard A, Ryder LP (1994). HLA and disease associations: detecting the strongest association.. Tissue Antigens.

[45] Oksenberg JR, Barcellos LF, Cree BA, Baranzini SE, Bugawan TL (2004). Mapping multiple sclerosis susceptibility to the HLA-DR locus in African Americans.. Am J Hum Genet.

[46] Maverakis E, Goodarzi H, Wehrli LN, Ono Y, Garcia MS (2011). The Etiology of Paraneoplastic Autoimmunity..

[47] Gilhus NE, Owe JF, Hoff JM, Romi F, Skeie GO (2011). Myasthenia gravis: a review of available treatment approaches..

