# Supplementary material for: Late Onset Myasthenia Gravis Is Associated with HLA DRB1*15:01 in the Norwegian Population
Source: PLoS One. 2012 May 9;7(5):e36603. doi: 10.1371/journal.pone.0036603 (PMC3348874; doi:10.1371/journal.pone.0036603)
Supplement: Table S1 — Allele frequencies in MG subgroups and controls. Allele groups that include rare alleles are: HLA-A*09 (23, 24), A*10 (25, 26, 34, 66), A*19 (29, 30, 31, 32, 33), A*28 (68, 69), B*05 (51, 52), B*12 (44, 45), B*15 (62), B*16 (38, 39), B*17 (57, 58), B*21 (49, 50), B*22 (54, 55, 56), B*40 (60, 61). (DOC) [file pone.0036603.s001.doc]

| **Locus**  **Table S1.** Allele frequencies in MG subgroups and controls | **Allele** | **Controls**  **n=652**  **N (freq)** | **EOMG**  **n=154**  **N (freq)** | **OR** | **P_nc_** | **LOMG**  **n=99**  **N (freq)** | **OR** | **P_nc_** | **MG 41-59**  **n=86**  **N (freq)** | **OR** | **P_nc_** | **Thymoma**  **n=30**  **N (freq)** | **OR** | **P_nc_** |
| --- | --- | --- | --- | --- | --- | --- | --- | --- | --- | --- | --- | --- | --- | --- |
| **HLA-A** | 01 | 206 (0.16) | 97 (0.31) | 2.4 | 6x10^-10^ | 25 (0.13) | 0.8 | 0.22 | 28 (0.16) | 0.8 | 0.90 | 7 (0.12) | 0.6 | 0.36 |
|  | 02 | 452 (0.35) | 75 (0.24) | 0.6 | 5x10^-4^ | 62 (0.31) | 0.9 | 0.38 | 49 (0.28) | 0.8 | 0.10 | 20 (0.33) | 0.9 | 0.83 |
|  | 03 | 201 (0.15) | 33 (0.11) | 0.7 | 0.04 | 38 (0.19) | 1.3 | 0.17 | 29 (0.16) | 1.1 | 0.60 | 11 (0.18) | 1.3 | 0.48 |
|  | 09 | 133 (0.10) | 22 (0.07) | 0.6 | 0.08 | 21 (0.10) | 1.1 | 0.82 | 14 (0.08) | 0.7 | 0.37 | 9 (0.15) | 1.2 | 0.26 |
|  | 10 | 53 (0.04) | 15 (0.05) | 1.2 | 0.48 | 11 (0.06) | 1.4 | 0.28 | 10 (0.06) | 1.5 | 0.23 | 5 (0.08) | 2.3 | 0.07 |
|  | 11 | 75 (0.06) | 12 (0.04) | 0.7 | 0.21 | 20 (0.10) | 1.9 | 0.02 | 12 (0.07) | 1.3 | 0.46 | 4 (0.07) | 1.3 | 0.60 |
|  | 19 | 129 (0.10) | 37 (0.12) | 1.3 | 0.26 | 14 (0.07) | 0.7 | 0.22 | 19 (0.11) | 1.1 | 0.59 | 2 (0.03) | 0.4 | 0.11 |
|  | 28 | 47 (0.04) | 17 (0.06) | 1.6 | 0.11 | 7 (0.04) | 1.0 | 0.94 | 11 (0.06) | 1.9 | 0.06 | 2 (0.03) | 1.9 | 0.85 |
| **HLA-B** | 05 | 39 (0.03) | 9 (0.03) | 0.9 | 0.85 | 4 (0.02) | 0.7 | 0.42 | 7 (0.04) | 1.3 | 0.46 | 3 (0.05) | 1.9 | 0.26 |
|  | 07 | 177 (0.15) | 39 (0.13) | 0.9 | 0.38 | 43 (0.22) | 1.6 | 0.01 | 29 (0.17) | 1.2 | 0.41 | 9 (0.16) | 1.1 | 0.78 |
|  | 08 | 164 (0.13) | 101 (0.33) | 3.1 | 1x10^-14^ | 12 (0.06) | 0.4 | 0.003 | 29 (0.17) | 1.3 | 0.22 | 3 (0.05) | 0.4 | 0.08 |
|  | 12 | 172 (0.14) | 24 (0.08) | 0.5 | 0.003 | 25 (0.12) | 0.9 | 0.55 | 15 (0.09) | 0.6 | 0.06 | 8 (0.14) | 1.0 | 0.99 |
|  | 13 | 16 (0.01) | 2 (0.01) | 0.6 | 0.39 | 4 (0.02) | 1.7 | 0.33 | 4 (0.02) | 1.9 | 0.20 | 1 (0.02) | 1.9 | 0.41 |
|  | 14 | 17 (0.01) | 4 (0.01) | 1.0 | 1.0 | 4 (0.02) | 1.6 | 0.38 | 2 (0.01) | 1.0 | 1.0 | 2 (0.03) | 3.0 | 0.09 |
|  | 15 | 136 (0.11) | 24 (0.08) | 0.7 | 0.08 | 16 (0.08) | 0.7 | 0.19 | 24 (0.14) | 1.3 | 0.26 | 6 (0.10) | 1.0 | 0.92 |
|  | 16 | 27 (0.02) | 5 (0.01) | 0.8 | 0.58 | 5 (0.03) | 1.2 | 0.68 | 3 (0.02) | 0.9 | 0.82 | 3 (0.05) | 2.7 | 0.07 |
|  | 17 | 41 (0.03) | 4 (0.01) | 0.4 | 0.06 | 5 (0.03) | 0.8 | 0.60 | 4 (0.02) | 0.8 | 0.55 | 1 (0.02) | 0.7 | 0.66 |
|  | 18 | 37 (0.03) | 9 (0.03) | 1.0 | 0.96 | 11 (0.06) | 1.9 | 0.06 | 5 (0.03) | 1.0 | 0.95 | 1 (0.02) | 0.8 | 0.77 |
|  | 21 | 18 (0.01) | 3 (0.01) | 0.7 | 0.57 | 2 (0.01) | 0.8 | 0.73 | 1(<0.01) | 0.6 | 0.44 | 1 (0.02) | 1.7 | 0.50 |
|  | 22 | 17 (0.01) | 3 (0.01) | 0.8 | 0.65 | 1 (<0.01) | 0.5 | 0.37 | 1(<0.01) | 0.6 | 0.49 | 3 (0.05) | 4.3 | 0.01 |
|  | 27 | 85 (0.07) | 15 (0.05) | 0.7 | 0.19 | 19 (0.10) | 1.4 | 0.19 | 7 (0.04) | 0.6 | 0.17 | 4 (0.07) | 1.0 | 0.88 |
|  | 35 | 86 (0.07) | 11 (0.04) | 0.5 | 0.03 | 22 (0.11) | 1.6 | 0.05 | 16 (0.08) | 1.4 | 0.26 | 4 (0.07) | 1.0 | 0.90 |
|  | 37 | 15 (0.01) | 4 (0.01) | 1.1 | 0.81 | 4 (0.02) | 1.8 | 0.27 | 1 (<0.01) | 0.7 | 0.60 | 3 (0.05) | 4.8 | 0.01 |
|  | 40 | 132 (0.11) | 48 (0.16) | 1.5 | 0.02 | 18 (0.09) | 0.8 | 0.45 | 22 (0.12) | 1.2 | 0.41 | 6 (0.10) | 1.0 | 0.99 |
|  | 41 | 6 (<0.01) | 0 | - | - | 1 (<0.01) | 0.7 | 0.99 | 0 | - | - | 0 | - | - |
|  | 42 | 1 (<0.01) | 0 | - | - | 1 (<0.01) | 6.0 | 0.07 | 0 | - | - | 0 | - | - |
|  | 47 | 5 (<0.01) | 1 (<0.01) | 1.0 | 0.91 | 1 (<0.01) | 1.7 | 0.54 | 0 | - | - | 0 | - | - |
|  | 48 | 4 (<0.01) | 0 | - | - | 0 | - | - | 0 | - | - | 0 | - | - |
|  | 70 | 1 (<0.01) | 0 | - | - | 0 | - | - | 0 | - | - | 0 | - | - |
| **HLA-C** | 01 | 50 (0.04) | 10 (0.03) | 0.9 | 0.68 | 5 (0.03) | 0.7 | 0.41 | 3 (0.02) | 0.5 | 0.20 | 3 (0.05) | 1.6 | 0.42 |
|  | 02 | 83 (0.06) | 12 (0.04) | 0.6 | 0.10 | 13 (0.07) | 1.0 | 0.87 | 7 (0.04) | 0.7 | 0.28 | 6 (0.10) | 1.8 | 0.16 |
|  | 03 | 278 (0.22) | 65 (0.21) | 1.0 | 0.92 | 33 (0.17) | 0.7 | 0.12 | 40 (0.24) | 1.1 | 0.53 | 11 (0.19) | 0.9 | 0.69 |
|  | 04 | 111 (0.08) | 18 (0.06) | 0.7 | 0.13 | 24 (0.12) | 1.5 | 0.09 | 19 (0.11) | 1.4 | 0.23 | 6 (0.10) | 1.3 | 0.52 |
|  | 05 | 127 (0.10) | 14 (0.05) | 0.5 | 0.005 | 13 (0.07) | 0.7 | 0.18 | 10 (0.06) | 0.6 | 0.11 | 4 (0.07) | 0.8 | 0.55 |
|  | 06 | 90 (0.07) | 16 (0.05) | 0.8 | 0.30 | 14 (0.07) | 1.0 | 0.83 | 9 (0.05) | 0.8 | 0.47 | 6 (0.10) | 1.6 | 0.24 |
|  | 07 | 414 (0.32) | 145 (0.47) | 1.9 | 6x10^-7^ | 68 (0.35) | 1.1 | 0.40 | 63 (0.37) | 1.3 | 0.19 | 15 (0.25) | 0.8 | 0.33 |
|  | 08 | 25 (0.02) | 5 (0.02) | 0.9 | 0.83 | 4 (0.02) | 1.2 | 0.75 | 2 (0.01) | 0.7 | 0.62 | 2 (0.03) | 2.2 | 0.21 |
|  | 12 | 41 (0.03) | 11 (0.04) | 1.2 | 0.64 | 7 (0.04) | 1.2 | 0.64 | 7 (0.04) | 1.4 | 0.41 | 2 (0.03) | 1.3 | 0.64 |
|  | 14 | 10 (0.01) | 2 (0.01) | 1.0 | 1.00 | 1(<0.01) | 0.9 | 0.94 | 3 (0.02) | 2.5 | 0.11 | 1 (0.02) | 3.2 | 0.14 |
|  | 15 | 17 (0.01) | 6 (0.02) | 1.6 | 0.31 | 3 (0.02) | 1.3 | 0.61 | 4 (0.02) | 1.9 | 0.19 | 1 (0.02) | 1.9 | 0.40 |
|  | 16 | 33 (0.03) | 2 (0.01) | 0.3 | 0.05 | 6 (0.03) | 1.3 | 0.54 | 3 (0.02) | 0.8 | 0.65 | 1 (0.02) | 0.8 | 0.98 |
|  | 17 | 9 (0.01) | 0 | - | - | 2 (0.01) | 1.8 | 0.40 | 0 | - | - | 0 | - | - |
|  | 18 | 0 | 0 | - | - | 1 (<0.01) | - | - | 0 | - | - | 0 | - | - |
| **HLA-DRB1** | 0101 | 132 (0.11) | 23 (0.07) | 0.7 | 0.11 | 20 (0.10) | 1.0 | 0.90 | 16 (0.09) | 0.9 | 0.67 | 5 (0.09) | 0.9 | 0.73 |
|  | 0102 | 5 (<0.01) | 3 (0.01) | 2.5 | 0.15 | 2 (0.01) | 2.9 | 0.14 | 0 | - | - | 0 | - | - |
|  | 0103 | 16 (0.01) | 5 (0.02) | 1.3 | 0.54 | 5 (0.03) | 2.1 | 0.12 | 3 (0.02) | 1.6 | 0.44 | 0 | - | - |
|  | 0201 | 0 | 0 | - | - | - | - | - | 1 (<0.01) | - | - | 0 | - | - |
|  | 0301 | 169 (0.13) | 97 (0.31) | 2.9 | 5x10^-13^ | 10 (0.05) | 0.4 | 0.001 | 24 (0.14) | 1.0 | 0.82 | 5 (0.09) | 0.7 | 0.32 |
|  | 0401 | 145 (0.12) | 25 (0.08) | 0.7 | 0.08 | 23 (0.12) | 1.0 | 0.95 | 18 (0.11) | 0.9 | 0.71 | 7 (0.12) | 1.1 | 0.82 |
|  | 0402 | 2 (<0.01) | 1 (<0.01) | 2.1 | 0.59 | 3 (0.02) | 9.5 | 0.02 | 0 | - | - | 0 | - | - |
|  | 0403 | 12 (0.10) | 1 (<0.01) | 0.5 | 0.33 | 1(<0.01) | 0.8 | 0.70 | 2 (0.01) | 1.5 | 0.56 | 1 (0.02) | 2.5 | 0.22 |
|  | 0404 | 94 (0.08) | 33 (0.11) | 1.5 | 0.07 | 18 (0.09) | 1.2 | 0.40 | 14 (0.08) | 1.1 | 0.70 | 3 (0.05) | 0.8 | 0.61 |
|  | 0405 | 3 (<0.01) | 0 | - | - | 4 (0.02) | 8.2 | 0.002 | 0 | - | - | 0 | - | - |
|  | 0407 | 3 (<0.01) | 4 (0.01) | 5.2 | 0.01 | 0 | - | - | 0 | - | - | 2 (0.03) | 14 | 0.01 |
|  | 0408 | 13 (0.01) | 4 (0.01) | 1.3 | 0.57 | 0 | - | - | 0 | - | - | 1 (0.02) | 2.4 | 0.26 |
|  | 0701 | 110 (0.09) | 11 (0.04) | 0.4 | 0.003 | 23 (0.12) | 1.4 | 0.18 | 15 (0.09) | 1.0 | 0.95 | 5 (0.09) | 1.0 | 0.91 |
|  | 0801 | 53 (0.04) | 4 (0.01) | 0.3 | 0.01 | 8 (0.04) | 1.0 | 0.99 | 3 (0.02) | 0.5 | 0.14 | 5 (0.09) | 2.3 | 0.07 |
|  | 0802 | 1 (<0.01) | 0 | - | - | 1 (<0.01) | 6.3 | 0.07 | 0 | - | - | 1 (0.02) | 21 | 0.06 |
|  | 0803 | 1 (<0.01) | 1 (<0.01) | 4.0 | 0.16 | 1 (<0.01) | 6.3 | 0.07 | 1 (<0.01) | 7.3 | 0.05 | 0 | - | - |
|  | 0804 | 0 | 0 | - | - | 0 | - | - | 0 | - | - | 0 | - | - |
|  | 0901 | 15 (0.01) | 4 (0.01) | 1.1 | 0.76 | 2 (0.01) | 1.0 | 0.98 | 8 (0.05) | 4.1 | 0.001 | 0 | - | 1 |
|  | 1001 | 9 (0.01) | 2 (<0.01) | 1.1 | 0.93 | 3 (0.02) | 2.3 | 0.15 | 2 (0.01) | 1.9 | 0.32 | 2 (0.03) | 5.7 | 0.01 |
|  | 1101 | 41 (0.03) | 5 (0.02) | 0.5 | 0.14 | 3 (0.02) | 0.5 | 0.21 | 3 (0.02) | 0.6 | 0.33 | 2 (0.03) | 1.3 | 0.69 |
|  | 1102 | 2 (<0.01) | 0 | - | - | 0 | - | - | 0 | - | - | 0 | - | - |
|  | 1103 | 4 (<0.01) | 1 (<0.01) | 1.0 | 0.99 | 0 | - | - | 0 | - | - | 0 | - | - |
|  | 1104 | 5 (<0.01) | 2 (<0.01) | 1.6 | 0.58 | 0 | - | - | 2 (0.01) | 2.6 | 0.24 | 0 | - | - |
|  | 1201 | 29 (0.02) | 5 (0.02) | 0.7 | 0.51 | 3 (0.02) | 0.7 | 0.45 | 6 (0.04) | 1.6 | 0.26 | 0 | - | - |
|  | 1301 | 92 (0.07) | 8 (0.03) | 0.4 | 3 x10^-3^ | 3 (0.03) | 0.2 | 4x10^-4^ | 5 (0.03) | 0.4 | 0.03 | 5 (0.09) | 1.3 | 0.59 |
|  | 1302 | 59 (0.04) | 20 (0.06) | 1.4 | 0.19 | 6 (0.03) | 0.7 | 0.33 | 12 (0.07) | 1.5 | 0.16 | 2 (0.03) | 0.9 | 0.82 |
|  | 1303 | 3 (<0.01) | 1 (<0.01) | 1.4 | 0.80 | 1(<0.01) | 2.1 | 0.54 | 1 (<0.01) | 2.1 | 0.48 | 0 | - | - |
|  | 1401 | 21 (0.02) | 6 (0.02) | 1.2 | 0.65 | 3 (0.02) | 1.0 | 0.97 | 6 (0.04) | 2.2 | 0.07 | 3 (0.05) | 3.6 | 0.02 |
|  | 1501 | 161 (0.13) | 39 (0.13) | 1.0 | 0.91 | 51 (0.26) | 2.4 | 2x10^-6^ | 25 (0.15) | 1.2 | 0.50 | 9 (0.16) | 1.3 | 0.49 |
|  | 1502 | 24 (0.02) | 1 (<0.01) | 0.2 | 0.05 | 1 (<0.01) | 0.3 | 0.19 | 2 (0.01 | 0.7 | 0.61 | 0 | - | - |
|  | 1601 | 3 (<0.01) | 1 (<0.01) | 1.7 | 0.53 | 0 | - | - | 1 (<0.01) | 31 | 0.19 | 0 | - | - |
|  | 1602 | 1 (<0.01) | 0 | - | - | 0 | - | - | 0 | - | - | 0 | - | - |
|  | 0810 | 0 | 0 | - | - | 1 (<0.01) | - | - | 0 | - | - | 0 | - | - |
|  | 1202 | 0 | 0 | - | - | 0 | - | - | 0 | - | - | 0 | - | - |
|  | 0305 | 1 (<0.01) | 0 | - | - | 0 | - | - | 0 | - | - | 0 | - | - |
|  | 0409 | 1 (<0.01) | 0 | - | - | 0 | - | - | 0 | - | - | 0 | - | - |
|  | 1417 | 1 (<0.01) | 0 | - | - | 0 | - | - | 0 | - | - | 0 | - | - |
|  | 1432 | 1 (<0.01) | 0 | - | - | 0 | - | - | 0 | - | - | 0 | - | - |
|  | 1504 | 1 (<0.01) | 0 | - | - | 0 | - | - | 0 | - | - | 0 | - | - |
|  | 1305 | 2 (<0.01) | 0 | - | - | 0 | - | - | 0 | - | - | 0 | - | - |
|  | 1407 | 1 (<0.01) | 0 | - | - | 0 | - | - | 0 | - | - | 0 | - | - |
|  | 1126 | 0 | 1 (<0.01) | - | - | 0 | - | - | 0 | - | - | 0 | - | - |
