# Supplementary material for: Late Onset Myasthenia Gravis Is Associated with HLA DRB1*15:01 in the Norwegian Population
Source: PLoS One. 2012 May 9;7(5):e36603. doi: 10.1371/journal.pone.0036603 (PMC3348874; doi:10.1371/journal.pone.0036603)
Supplement: Table S2 — Clinical characteristics of the MG study cohort (n = 339) in relation to the most strongly associated HLA genotypes of this study. 1According to MGFA classification [39]: ocular MG (MGFA grade I), generalised MG (MGFA grade II-V), not available in 12 cases. 2Thymus histopathology: hyperplasia or normal/atrophy, not available in 26 cases. 3Concomitant immune-mediated diseases in patients include thyroid disease (hypo-, hyperthyroidsm, thyroiditis), type 1-diabetes, rheumatic diseases, systemic lupus erythematosus (SLE), celiac disease, inflammatory bowel diseases (Crohńs disease or ulcerative colitits). *MG patients with thymoma (n = 30) were excluded. − = positives, − = negatives. (DOC) [file pone.0036603.s002.doc]

**Table S2.** Clinical characteristics of the MG study cohort (n=339) in relation to the most strongly associated HLA genotypes of this study

| HLA genotype  (n= patients)* | AChR-ab+  n (%) | Female MG n (%) | EOMG    n (%) | Ocular MG^1^  n (%) | Thymectomy  n (%) | Thymus hyperplasia^2^  n (%) | Immune-mediated diseases^3^  n (%) |
| --- | --- | --- | --- | --- | --- | --- | --- |
| B*08+/ DRB1*X  (n=130) | 107 (82) | 105 (81) | 93 (72) | 21 (16) | 78 (60) | 46 (59) | 44 (34) |
| B*08+/ DRB1*03:01+  (n=112) | 89 (79) | 91 (81) | 80 (71) | 18 (16) | 66 (59) | 39 (59) | 41 (37) |
| B*08-/ DRB1*X  (n=209) | 163 (78) | 110 (53) | 61 (29) | 44 (21) | 61 (29) | 23 (38) | 64 (31) |
| B*08-/ DRB1*15:01+ (n=83) | 68 (82) | 46 (55) | 21 (25) | 16 (19) | 19 (23) | 8 (42) | 26 (31) |
